# Acute heat stress-indued apoptosis in mouse skeletal muscle is not associated with alteration of glutamine homeostasis

**DOI:** 10.1371/journal.pone.0278176

**Published:** 2022-11-28

**Authors:** Yifan Chen, Tianzheng Yu, Patricia A. Deuster

**Affiliations:** 1 Consortium for Health and Military Performance, Department of Military and Emergency Medicine, Uniformed Services University, Bethesda, MD, United States of America; 2 Henry M Jackson Foundation for the Advancement of Military Medicine, Bethesda, MD, United States of America; Hunan Agricultural University, CHINA

## Abstract

We previously demonstrated that exposing mice to heat causes functional and ultrastructural mitochondrial alterations and apoptosis in skeletal muscle. Emerging evidence indicates that glutamine (Gln) deprivation may increase cell susceptibility to apoptosis whereas Gln supplementation may protect cells against heat stress. In this study, we investigated the effect of short-term Gln treatment on heat-induced changes in mouse skeletal muscle. Male mice received vehicle, low-dose Gln (100 mg/kg/d) or high-dose Gln (300 mg/kg/d) through daily gavage for 10 days before a heat exposure test. During heat exposure, mice displayed a hyperthermic response and no significant differences in peak core body temperature were noted across the three groups. Neither heat exposure nor pretreatment with low-dose or high-dose Gln significantly affected Gln concentrations in plasma and gastrocnemius muscles. Heat-exposed mice had significantly higher caspase 3/7 levels in gastrocnemius muscle compared to unexposed controls. Heat exposure significantly increased ROS production and mitochondrial fragmentation and decreased mitochondrial membrane potential in flexor digitorum brevis muscle. These changes were not affected by low- or high-dose Gln pretreatment. Together, acute heat stress did not disrupt Gln homeostasis in mouse skeletal muscle and Gln supplementation did not protect mouse skeletal muscle against heat-induced injury. The results of this study do not support a role of Gln in heat-induced skeletal muscle apoptosis.

## Introduction

Glutamine (Gln) is the most abundant free amino acid in the body and serves as a substrate for the biosynthesis of proteins and other molecules to maintain cellular integrity and function [1–4]. Although Gln is a nonessential amino acid, it becomes conditionally essential in some catabolic situations such as prolonged starvation, sepsis and long-duration physical exercise [3]. Gln deprivation can result in an increased susceptibility to cell stress and apoptosis [5, 6]. On the other hand, Gln supplementation may reduce complication rates in critically ill and postoperative patients [7] and mitigate numerous risk factors for various pathophysiological conditions [4, 8].

Exposure to high temperature may cause an increase in core body temperature (Tc) or heat stress, and lead to organ dysfunction or injury. Gln is known to play an important role in maintaining cell and tissue integrity [4] and thus the use of Gln to prevent the detrimental effects of heat stress on various organs has gained attention. The intestinal barrier is particularly susceptible to injury under heat stress conditions [9] such that its integrity and function can be compromised [10, 11]. Increased intestinal barrier permeability, which allows permeation of luminal antigens and endotoxins into the circulation, is believed to contribute to heat-induced hyperthermic response and injury [12, 13]. Intestinal epithelial cells use Gln as their principal metabolic fuel [10]. Thus, Gln protection of the intestinal barrier has been studied in a variety of heat stress models. Gln treatment has been shown to prevent heat-induced injury in *in vitro* intestinal epithelial cells [14, 15] and heat -induced intestinal barrier dysfunction in mice [16], rats [17] and humans [18, 19].

Skeletal muscle is also one of the organs susceptible to heat injury. Animal studies by our laboratory and others have shown that heat exposure causes oxidative stress, mitochondrial dysfunction and apoptosis in skeletal muscle [20–23]. Skeletal muscle is quantitatively the most relevant site of Gln stock, synthesis, and release [3] and also consumes significant amount of Gln under stressful conditions such as exercise [24]. Gln homeostasis is essential for skeletal muscle health and performance. We have previously demonstrated that Gln depletion compromises mitochondrial function and cell viability in C2C12 skeletal muscle cells under heat stress [25]. In addition, animal and human studies have demonstrated that Gln supplementation can potentially reduce muscle fatigue and damage during prolonged exercise [2, 26]. There is little information on how heat stress affects skeletal muscle Gln homeostasis and whether Gln supplementation protects skeletal muscle against the adverse effects of heat stress. Therefore, the current study aimed to investigate the effect of heat exposure on Gln and the effect of short-term (10 days) Gln supplementation on heat-induced apoptosis in the skeletal muscle of mice. We hypothesized that administration of Gln to mice would prevent skeletal muscle injury during heat exposure.

## Materials and methods

### Animals

All procedures were approved by the Uniformed Services University Institutional Animal Care and Use Committee (protocol number: MEM-18-059). Male C57BL/6J Mice (6 weeks old) were purchased from Jackson Laboratories (Bar Harbor, ME) and maintained in a temperature-controlled (22°C) animal facilities at the Uniformed Services University, with 12-hour light/dark cycle and free access to food & water. One week after arrival, mice were surgically implanted with abdominal temperature transponders (BioTherm13, Biomark Inc, Boise, ID) under anesthesia with isoflurane (4% induction, 2% maintenance) [27]. Immediately following the surgical procedures, mice each received a single subcutaneous injection of buprenorphine SR (0.5 mg/kg). After two weeks of recovery, mice were randomly assigned to one sham control and three experimental groups (*n* = 6 per group). Mice in experimental groups were administered vehicle (water), 100 mg Gln or 300 mg Gln/kg body weight by oral gavage once a day for 10 days. L-Gln was purchased from Sigma-Aldrich, St Louis, MO. On day 11, experimental mice underwent a heat exposure experiment as previously described [28]. Briefly, mice were acclimated in an environmental chamber at 22°C (Model 3950, Thermo Forma, Marietta, OH) the night before heat exposure experiments and their core body temperature (Tc) and activity were continuously recorded. Heat exposure began the following morning after stable baseline data were obtained. Food and water were removed from cages before exposure. The heating element in the chamber was turned on (preset temperature of 39.5°C) for 180 minutes. Immediately after heat exposure, mice were anesthetized with isoflurane for terminal blood and tissue collection. Sham controls were maintained at room temperature during the entire period. All mice were 10–12 weeks old on the days of tissues collection.

### Blood and muscle sample processing

Blood was collected through cardia puncture and transferred into heparin-coated tubes. Following centrifugation, aliquots of plasma were frozen at—80°C before analysis. Mouse flexor digitorum brevis (FDB) myofibers were isolated by digestive enzymes, 3 mg/ml collagenase A and 1 mg/ml dispase II (11088793001 and D4693, Sigma-Aldrich, St Louis, MO) [29]. Isolated myofibers were resuspended in Dulbecco’s modified eagle medium (DMEM) containing 0.2% bovine serum albumin (BSA) and 0.1% gentamycin. Mouse gastrocnemius muscles were removed and snap frozen in optimal cutting temperature (OCT) compound for cryostat sectioning.

### Gln concentration

Concentrations of Gln in plasma and gastrocnemius muscle were measured using a colorimetric assay kit (ab197011, Abcam, Waltham, MA) per the manufacture’s instruction. Briefly, plasma samples and homogenized gastrocnemius muscle were centrifugated at 10,000 X g, 4°C, for 5 and10 min, respectively. The supernatants were transferred to a 96-well plate before incubation with the hydrolysis and development enzyme mix. Absorbance was measured at 450 nm in a microplate reader.

### Caspase 3/7 activity

Cryocut sections (5 μm) OCT-embedded frozen gastrocnemius muscle blocks were mounted on superfrost plus slides. Caspase activity was measured with CellEvent™ Caspase-3/7 Green detection reagent (ThermoFisher Scientific, Waltham, MA). Fluorescence images were acquired by using an imaging system consisting of a Nikon Eclipse Ti epifluorescence microscope and digital camera (Melville, NY). Excitation and emission wavelengths were 480/535 nm. Quantitative analysis was performed using the NIH ImageJ software.

### Reactive oxygen species (ROS), mitochondrial morphology and membrane potential

Mouse FDB myofibers were incubated with 5 μM dihydroethidium (DHE, Thermo Fisher Scientific, Waltham, MA) for 15 min, washed by centrifugation and resuspended in PBS. ROS levels were determined using fluorescence imaging microscopy of DHE (excitation/emission: 555/613 nm). To visualize mitochondria and mitochondrial membrane potential (ΔΨm), mouse FDB myofibers were incubated with 100 nM MitoTracker Red CMXRos and 100 nM tetramethylrhodamine ethyl ester (TMRE, Thermo Fisher Scientific, Waltham, MA) for 15 min. Mitochondrial fragmentation was quantified as the percentage of myofibers containing 10 or more mitochondria with length less than 3 μm. Mitochondrial membrane potential was measured by using fluorescence imaging microscopy of TMRE (excitation/emission: 555/613 nm). The intensity of fluorescence images was quantified by using the NIH ImageJ software.

### Statistical analysis

All data are presented as mean ± SD. Statistical analyses were performed by using the GraphPad Prism 9 (GraphPad Software, La Jolla, CA). Statistical analysis was performed with two-tailed Student’s t test or one-way analysis of variance followed by Tukey’s multiple comparison test. Values of p < 0.05 were considered statistically significant.

## Results

**Fig 1:**
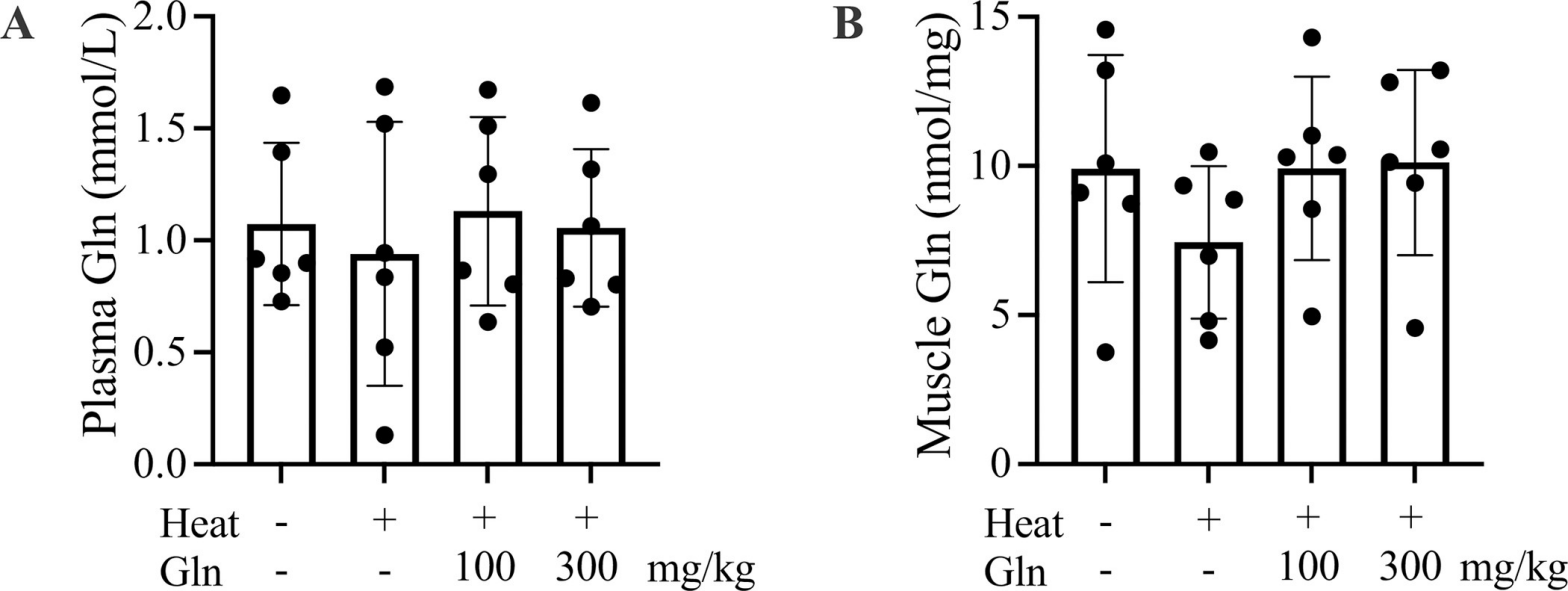
Gln concentrations in plasma (A) and gastrocnemius muscle (B) of sham or heat-exposed mice. Values are mean ± SD. * p < 0.05, n = 6 mice per group.

There were no significant differences in the animal’s final body weight or their baseline Tc between the groups (Table 1). During heat exposure, Tc was significantly elevated in mice pretreated with vehicle, 100 mg/kg or 300 mg/kg Gln, when compared to the baseline (p < 0.0001). No significant differences in peak Tc were found between the three groups. Heat exposure did not affect Gln concentrations in plasma (Fig 1A) and gastrocnemius muscle (Fig 1B). No significant changes in plasma and muscle Glu levels were detected in mice pretreated with 100 mg/kg Gln or 300 mg/kg Gln compared to vehicle groups.

**Table 1:** Body weight and Tc of sham and heat-exposed mice.

	Sham		Heat-exposed	
		vehicle	100 mg/kg Gln	300 mg/kg Gln
weight (g)	23.8 ± 1.2	24.0 ± 1.2	23.9 ± 1.5	24.3 ± 0.5
baseline Tc (C°)	36.6 ± 0.6	36.3 ± 0.7	36.1 ± 1.1	36.3 ± 1.0
peak Tc (C°)		40.9 ± 0.6 *	41.2 ± 0.7 *	41.1 ± 0.5 *

n = 6 per group.

* p < 0.0001 versus baseline Tc.

**Fig 2:**
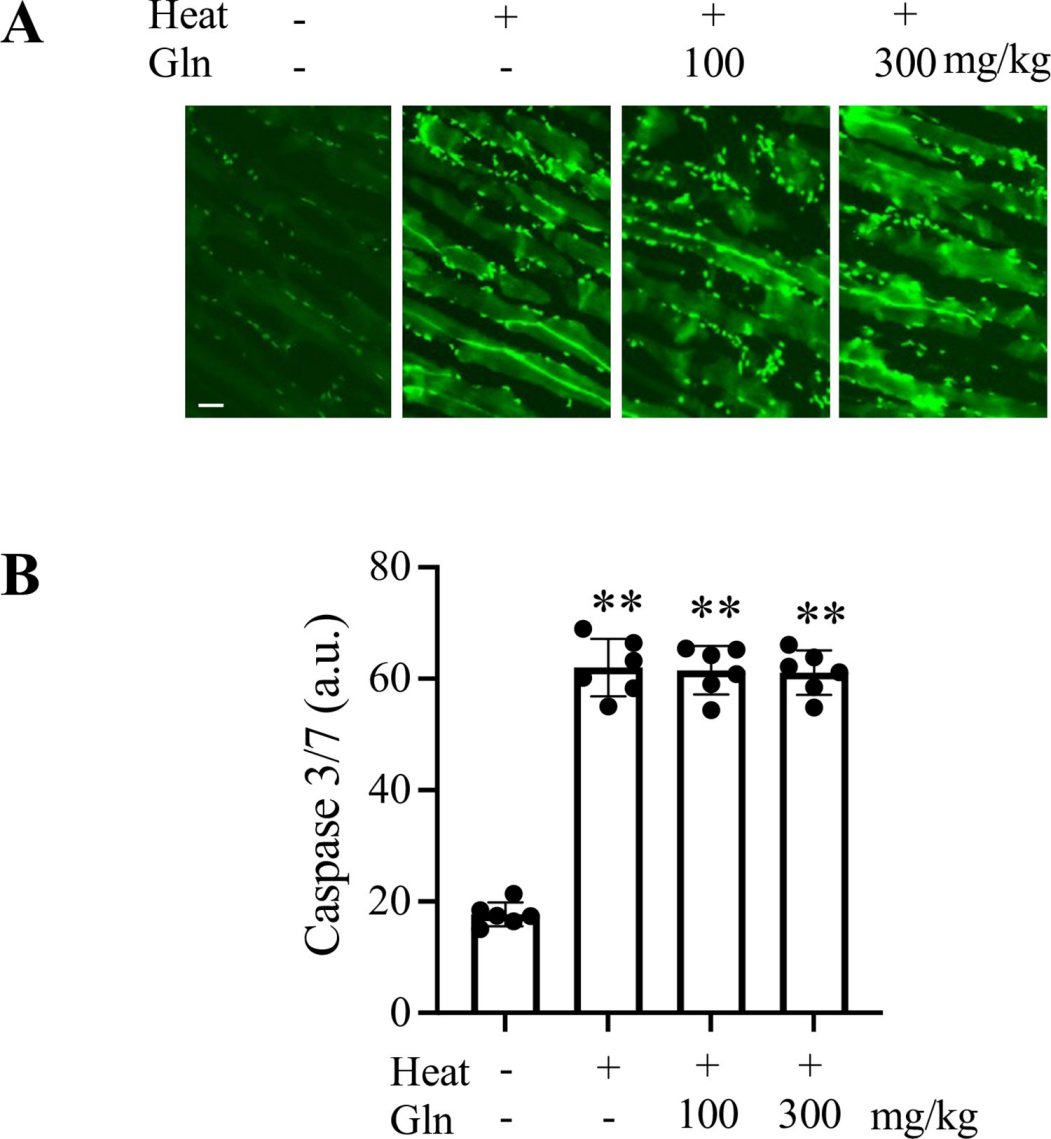
Activation of caspase 3/7 in gastrocnemius muscle of sham and heat-exposed mice. A: Representative images show CellEvent Caspase 3/7-labeled myofibers from each group (scale bar: 20 μm). B: Fluorescence intensities were quantified on randomly selected 20 fields per animal and expressed in arbitrary units (a.u.). Values are mean ± SD. ** p < 0.0001, n = 6 mice per group.

Heat stress can lead to skeletal muscle apoptosis [21, 22] and Gln can produce an anti-apoptotic effect by inhibiting activation of caspases [1]. Accordingly, we determined levels of caspase 3/7 activity in mouse gastrocnemius muscle tissues (Fig 2). Activity of caspase 3/7 was approximately 3-fold higher in heat-exposed mice than in sham controls (p < 0.0001). Pretreatment with 100 mg/kg or 300 mg/kg Gln had no effect on heat exposure-induced activation of caspase 3/7.

**Fig 3:**
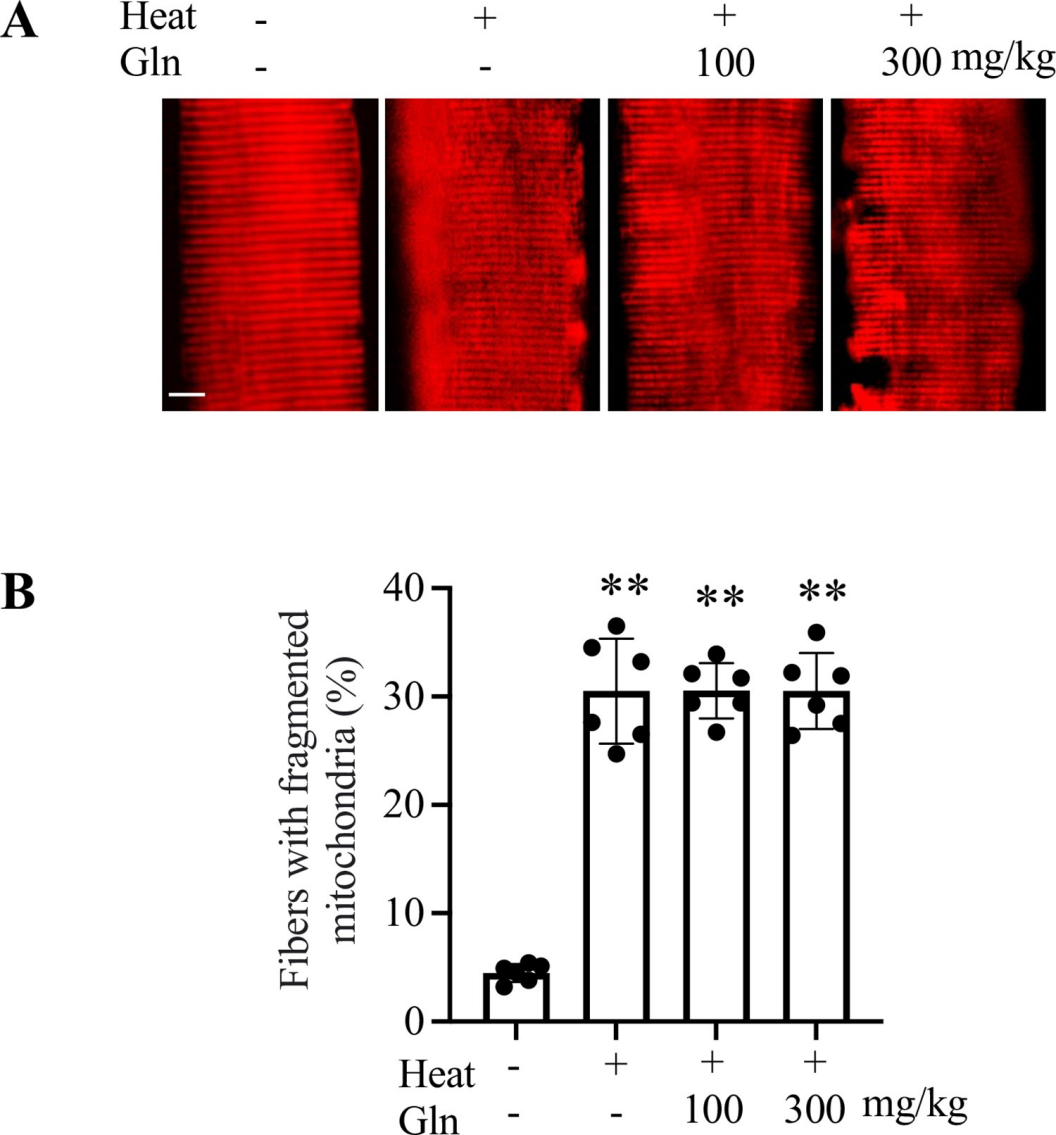
Mitochondrial morphology in flexor digitorum brevis muscle of sham and heat-exposed mice. A: Representative images show MitoTracker-stained myofibers from each group (scale bar: 10 μm). B: Quantitative analysis of mitochondrial morphology was performed on randomly selected 20 myofibers per animal. Values are mean ± SD. ** p < 0.0001, n = 6 mice per group.

**Fig 4:**
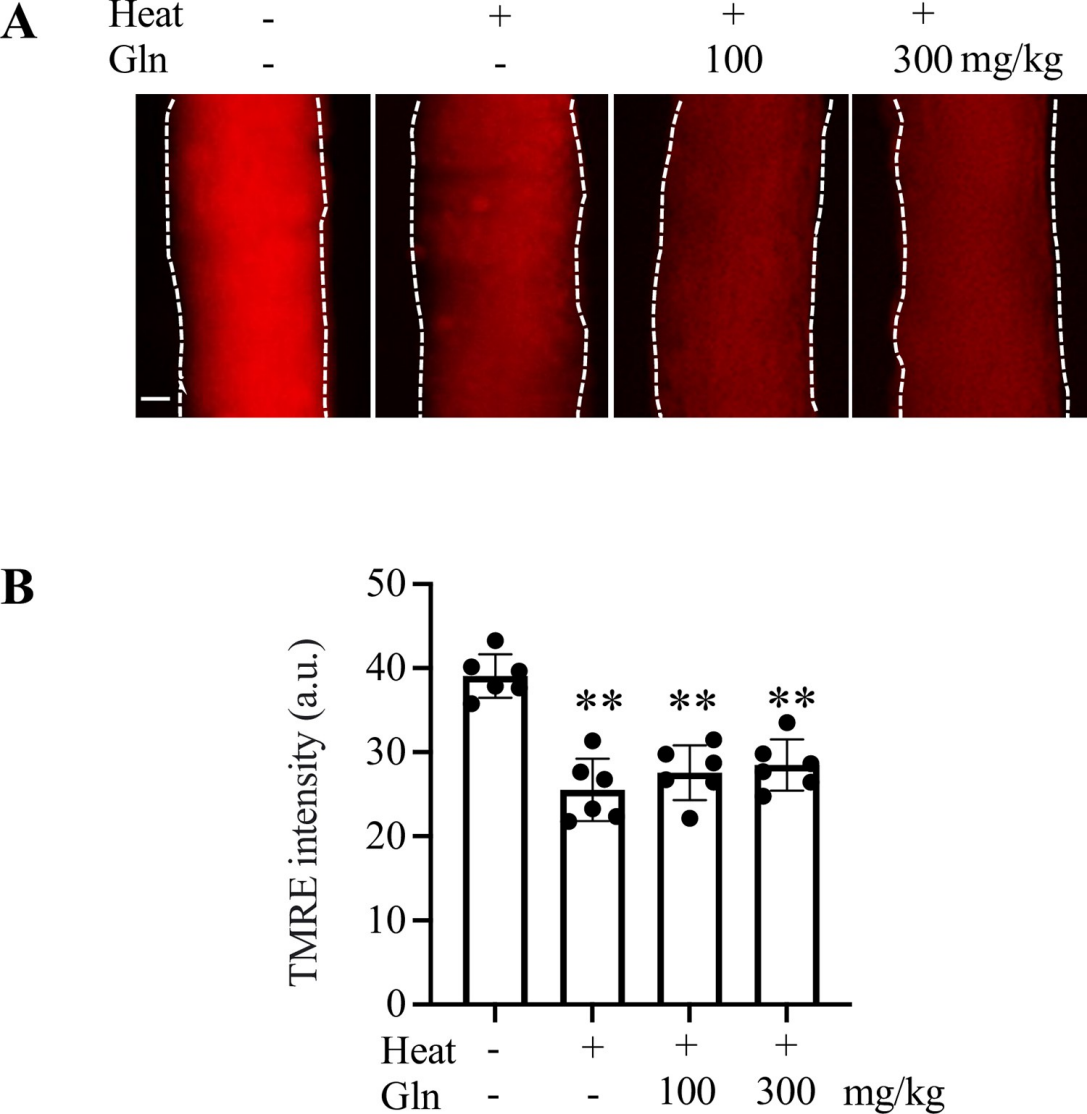
Mitochondrial membrane potential in flexor digitorum brevis muscle of sham and heat-exposed mice. A: Representative images show TMRE-stained myofibers from each group (scale bar: 10 μm). B: Fluorescence intensities were quantified on randomly selected 20 myofibers per animal and expressed in arbitrary units (a.u.). Values are mean ± SD. ** p < 0.0001, n = 6 mice per group.

Mitochondrial fission and dysfunction play a role in heat-induced skeletal muscle apoptosis [21, 22]. Maintaining Gln homeostasis is important for the protection of mitochondrial integrity and function against heat stress [25]. We examined mitochondrial morphology and membrane potential in mouse FDB myofibers. An ~6-fold increase in mitochondrial fragmentation (short tubular or punctate mitochondria) was observed in heat-exposed mice when compared to sham controls (p < 0.0001, Fig 3). The heat-induced fragmentation of mitochondria was not affected by pretreatment with 100 mg/kg or 300 mg/kg Gln. Heat exposure decreased mitochondrial membrane potential by 27–35% in mouse FDB myofibers (p < 0.0001, Fig 4). This heat-induced loss of mitochondrial membrane potential was not prevented by pretreatment with 100 mg/kg or 300 mg/kg Gln.

**Fig 5:**
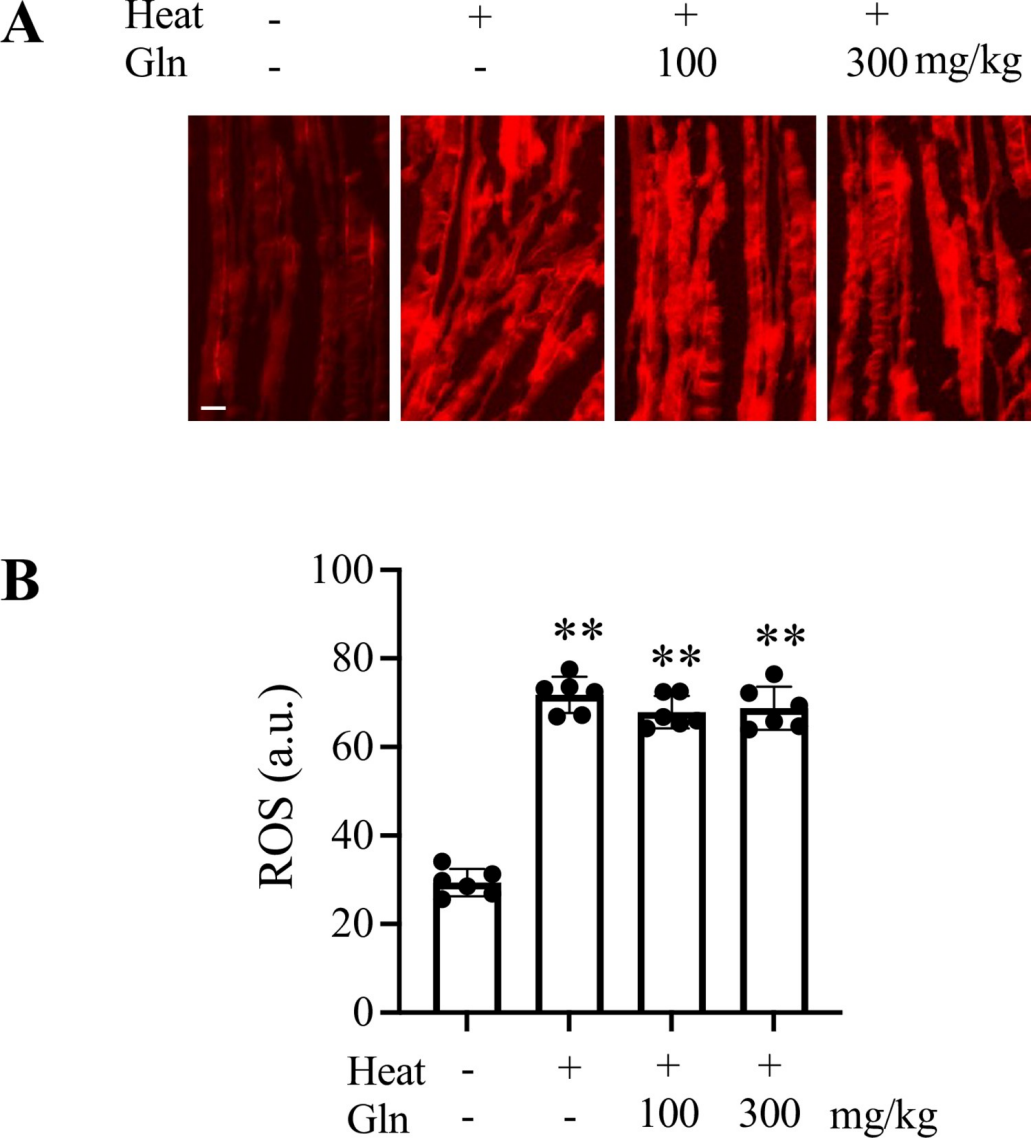
Generation of ROS in flexor digitorum brevis muscle of sham and heat-exposed mice. A: Representative images show DHE-stained myofibers from each group (scale bar: 20 μm). B: Fluorescence intensities were quantified on randomly selected 20 myofibers per animal and expressed in arbitrary units (a.u.). Values are mean ± SD. ** p < 0.0001, n = 6 mice per group.

Oxidative stress has been linked to heat-induced mitochondrial dysfunction in skeletal muscle [30, 31]. Gln can improve antioxidant capacity [1, 32]. We measured levels of ROS in mouse FDB myofibers (Fig 5). The production of ROS was ~2.5-fold higher in heat-exposed mice than in sham controls (p < 0.0001). Pretreatment with 100 mg/kg or 300 mg/kg Gln had no effect on this heat-induced generation of ROS.

## Discussion

In contrast to our hypothesis, the results of this study do not support a protective effect of Gln supplementation on mouse skeletal muscle against acute heat stress. We showed that pretreatment of mice with Gln neither affected Gln homeostasis in the skeletal muscle under heat stress, nor prevented heat-induced skeletal muscle alterations. Although heat exposure did result in significant heat stress or hyperthermia, it did not affect Gln levels of plasma or skeletal muscle in mice. Collectively, this study does not support a role for Gln in the regulation of heat stress response in mouse skeletal muscle.

Gln is not only a major respiratory fuel for many cell types [3], but also contributes to a wide range of cellular processes [4]. In particular, its modulatory role in stress responses [1, 9, 33, 34] prompted our interest in its potential application for the prevention of injury from heat stress. The heat-shock response or the rapid synthesis of heat shock proteins (HSP) is an important cellular defense mechanism against various insults. These proteins function as intracellular chaperones to enable organisms to cope with thermal damage to proteins, nucleic acids, and membranes. It has been demonstrated *in vitro* and *in vivo* that Gln enhances heat stress induction of cellular HSP [9, 17, 34]. There is evidence linking anti-heat stress effects of Gln to the induction of HSPs. In cell line models of acute heat stress, Gln treatment increases cellular HSP levels and yields cytoprotective effects [14, 35]. In rats, Gln supplementation increases HSP70 levels in intestinal tissues, reduces hyperthermia-induced intestinal barrier permeability and improves post-hyperthermia survival [17]. However, clinical trials regarding the effects of Gln supplementation on gut function during exercise in the heat have produced inconsistent findings [18, 36]. Supplemental Gln two hours before exercise in 30°C prevents gut permeability in a dose-dependent manner (0.25, 0.5, and 0.9 g/kg of fat free mass), compared to the placebo [18]. In contrast, consumption of supplemental Gln (0.9 g/kg of fat free mass) one hour before exercise in 35°C is ineffective in preventing elevations in intestinal damage makers and inflammatory cytokines, and improving exercise performance [36]. Gln’s protective benefit against acute heat stress is also complicated by its limited effect on reducing an increase in body temperature. Previous studies have shown that although preventing intestinal barrier dysfunction, Gln supplementation appears to produce a brief attenuating effect on hyperthermic response during passive heat exposure in mice [16] and does not affect body temperature elevation during exertional heat stress in humans [18, 19]. It is unclear whether these inconsistent results are attributable to the differences in body temperature elevation in the mouse model of passive heat exposure (~40.5°C) [16] versus human models of exertional heat stress (38.5–39.5°C) [18, 19]. Here we demonstrated that pretreatment with either dose of Gln does not significantly affect heat-induced hyperthermia (Table 1). We acknowledge the limitation of not assessing the intestinal function in this study. Our results revealed no anti-hyperthermic effect of Gln pretreatment against acute heat stress.

Growing evidence suggests that Gln is also involved in the regulation of apoptosis [1, 4], which can be triggered by heat stress [15, 21]. In *in vitro* experiments, Gln deprivation causes apoptotic cell death [37], whereas Gln treatment increases cell survival and prevents apoptotic cell death against heat stress [15]. The molecular mechanisms by which Gln regulates heat stress-induced apoptosis are not fully defined and seem to be complex and multifactorial. Gln may directly or indirectly affect activation of the caspases responsible for heat shock-induced cell death. Gln deprivation has been shown to cause activation of caspases 2 and 3 [37]. Activation of caspase 2 [38] or caspase 3 [15, 39] is an essential event during heat-induced apoptosis. Gln seems to exert an inhibitory effect on activation of caspase 2 [1] and caspase 3 [14, 15] by acting on redox signaling [1] as well as on PI3-K/Akt and MAPK pathways upstream of caspase activation [15]. In particular, Gln has been shown to enhance activation of ERK1/2 and Akt and prevent activation of p38-MAPK, which lowers caspase 3 activation, in *in vitro* cells in response to heat-stress [15]. Furthermore, administration of Gln prevented heat stress induced decreases in expression and signaling of cellular epidermal growth factor receptors, which resulted in enhanced ERK1/2 and decreased p38-MAPK signaling [15]. To our knowledge, the present study is the first to investigate the effects of Gln on heat-induced apoptosis *in vivo*. Our results consistently demonstrate that heat exposure causes oxidative stress, mitochondrial dysfunction and caspase 3/7 activation in mouse skeletal muscle. These changes are not affected by either dose of Gln. Thus, the findings of this study indicate that Gln supplementation produces no anti-apoptotic effect in mouse skeletal muscle against acute heat stress.

The current study also demonstrated that neither heat exposure nor 10-day supplementation of 100 mg/kg or 300 mg/kg Gln affected plasma and muscle Gln levels in mice compared to sham mice with vehicle. In our preliminary study, we also found that plasma and muscle Gln levels did not differ in sham groups pre-treated with Gln (300 mg/kg, n = 3, data not shown) vs vehicle. Similar findings have been observed in rats treated with a higher Gln dose (1 g/kg/day) for a longer duration (21 days) [40]. These results suggest that a marked increase in Gln intake does not disrupt its homeostasis in the body. In this study, we also found no alterations in plasma and muscle Gln following heat exposure. There is little information available in the literature concerning the effects of heat stress on Gln in the body. Likewise, Gln homeostasis does not seem to alter under other acute stress conditions such as prolonged or high-intensity exercise. For example, no changes in plasma or skeletal muscle Gln are observed in rats following two-hour swimming exercise [26]. The subjects taking a Gln supplement show no improvement in their 20-km cycling time trials in the heat (35°C) [36]. The results of this study also demonstrated that Gln supplementation provide no protection against acute heat stress. Together, it seems that during a single bout of heat exposure or exercise the physiological requirement for Gln may not exceed the capacity for its endogenous synthesis in skeletal muscle. This may in part explain why in this study Gln supplementation is ineffective mitigating the adverse effects of heat stress on mouse skeletal muscle.

## Conclusion

In summary, the main findings of this study were that acute heat stress does not alter Gln homeostasis nor does Gln serve a role in heat-induced apoptosis in mouse skeletal muscle. These results suggest that during acute heat stress Gln may not become conditionally essential. Future studies are needed to investigate whether the application of Gln is beneficial against prolonged heat stress in other tissues.

## References

[1] Matés JM, Segura JA, Alonso FJ, Márquez J. Pathways from glutamine to apoptosis. Front Biosci. 2006;11(3):3164–80. Epub 20060901. doi: 10.2741/2040 . 16720383

[2] Coqueiro AY, Rogero MM, Tirapegui J. Glutamine as an Anti-Fatigue Amino Acid in Sports Nutrition. Nutrients. 2019;11(4). Epub 20190417. doi: 10.3390/nu11040863 ; PubMed Central PMCID: PMC6520936. 30999561 PMC6520936

[3] Cruzat V, Macedo Rogero M, Noel Keane K, Curi R, Newsholme P. Glutamine: Metabolism and Immune Function, Supplementation and Clinical Translation. Nutrients. 2018;10(11). Epub 20181023. doi: 10.3390/nu10111564 ; PubMed Central PMCID: PMC6266414. 30360490 PMC6266414

[4] Durante W. The Emerging Role of l-Glutamine in Cardiovascular Health and Disease. Nutrients. 2019;11(9). Epub 20190904. doi: 10.3390/nu11092092 ; PubMed Central PMCID: PMC6769761. 31487814 PMC6769761

[5] Exner R, Weingartmann G, Eliasen MM, Gerner C, Spittler A, Roth E, et al. Glutamine deficiency renders human monocytic cells more susceptible to specific apoptosis triggers. Surgery. 2002;131(1):75–80. doi: 10.1067/msy.2002.118318 . 11812966

[6] Fumarola C, Zerbini A, Guidotti GG. Glutamine deprivation-mediated cell shrinkage induces ligand-independent CD95 receptor signaling and apoptosis. Cell Death Differ. 2001;8(10):1004–13. doi: 10.1038/sj.cdd.4400902 . 11598798

[7] Novak F, Heyland DK, Avenell A, Drover JW, Su X. Glutamine supplementation in serious illness: a systematic review of the evidence. Crit Care Med. 2002;30(9):2022–9. doi: 10.1097/00003246-200209000-00011 . 12352035

[8] Sadaf A, Quinn CT. L-glutamine for sickle cell disease: Knight or pawn? Exp Biol Med (Maywood). 2020;245(2):146–54. Epub 20200127. doi: 10.1177/1535370219900637 ; PubMed Central PMCID: PMC7016414. 31985279 PMC7016414

[9] Iwashita Y, Sakiyama T, Musch MW, Ropeleski MJ, Tsubouchi H, Chang EB. Polyamines mediate glutamine-dependent induction of the intestinal epithelial heat shock response. American journal of physiology Gastrointestinal and liver physiology. 2011;301(1):G181–7. Epub 20110421. doi: 10.1152/ajpgi.00054.2011 ; PubMed Central PMCID: PMC3129932. 21512157 PMC3129932

[10] King MA, Rollo I, Baker LB. Nutritional considerations to counteract gastrointestinal permeability during exertional heat stress. Journal of applied physiology. 2021;130(6):1754–65. Epub 2021/05/07. doi: 10.1152/japplphysiol.00072.2021 . 33955260

[11] Larson SD, Li J, Chung DH, Evers BM. Molecular mechanisms contributing to glutamine-mediated intestinal cell survival. American journal of physiology Gastrointestinal and liver physiology. 2007;293(6):G1262–71. Epub 20071004. doi: 10.1152/ajpgi.00254.2007 ; PubMed Central PMCID: PMC2432018. 17916648 PMC2432018

[12] Dokladny K, Zuhl MN, Moseley PL. Intestinal epithelial barrier function and tight junction proteins with heat and exercise. Journal of applied physiology. 2016;120(6):692–701. Epub 2015/09/12. doi: 10.1152/japplphysiol.00536.2015 ; PubMed Central PMCID: PMC4868372. 26359485 PMC4868372

[13] Szymanski MC, Gillum TL, Gould LM, Morin DS, Kuennen MR. Short-term dietary curcumin supplementation reduces gastrointestinal barrier damage and physiological strain responses during exertional heat stress. Journal of applied physiology. 2018;124(2):330–40. Epub 2017/09/25. doi: 10.1152/japplphysiol.00515.2017 . 28935827

[14] Kallweit AR, Baird CH, Stutzman DK, Wischmeyer PE. Glutamine prevents apoptosis in intestinal epithelial cells and induces differential protective pathways in heat and oxidant injury models. JPEN Journal of parenteral and enteral nutrition. 2012;36(5):551–5. Epub 20120427. doi: 10.1177/0148607112445579 . 22544840

[15] Niederlechner S, Baird C, Petrie B, Wischmeyer E, Wischmeyer PE. Epidermal growth factor receptor expression and signaling are essential in glutamine’s cytoprotective mechanism in heat-stressed intestinal epithelial-6 cells. American journal of physiology Gastrointestinal and liver physiology. 2013;304(5):G543–52. Epub 20121228. doi: 10.1152/ajpgi.00418.2012 ; PubMed Central PMCID: PMC3602678. 23275616 PMC3602678

[16] Soares AD, Costa KA, Wanner SP, Santos RG, Fernandes SO, Martins FS, et al. Dietary glutamine prevents the loss of intestinal barrier function and attenuates the increase in core body temperature induced by acute heat exposure. Br J Nutr. 2014;112(10):1601–10. Epub 2014/10/18. doi: 10.1017/S0007114514002608 . 25322775

[17] Singleton KD, Wischmeyer PE. Oral glutamine enhances heat shock protein expression and improves survival following hyperthermia. Shock. 2006;25(3):295–9. doi: 10.1097/01.shk.0000196548.10634.02 . 16552363

[18] Pugh JN, Sage S, Hutson M, Doran DA, Fleming SC, Highton J, et al. Glutamine supplementation reduces markers of intestinal permeability during running in the heat in a dose-dependent manner. Eur J Appl Physiol. 2017;117(12):2569–77. Epub 20171020. doi: 10.1007/s00421-017-3744-4 ; PubMed Central PMCID: PMC5694515. 29058112 PMC5694515

[19] Zuhl MN, Lanphere KR, Kravitz L, Mermier CM, Schneider S, Dokladny K, et al. Effects of oral glutamine supplementation on exercise-induced gastrointestinal permeability and tight junction protein expression. Journal of applied physiology. 2014;116(2):183–91. Epub 20131127. doi: 10.1152/japplphysiol.00646.2013 ; PubMed Central PMCID: PMC3921361. 24285149 PMC3921361

[20] Zuo L, Christofi FL, Wright VP, Liu CY, Merola AJ, Berliner LJ, et al. Intra- and extracellular measurement of reactive oxygen species produced during heat stress in diaphragm muscle. AmJPhysiol Cell Physiol. 2000;279(4):C1058–C66. doi: 10.1152/ajpcell.2000.279.4.C1058 11003586

[21] Yu T, Deuster P, Chen Y. Role of dynamin-related protein 1-mediated mitochondrial fission in resistance of mouse C2C12 myoblasts to heat injury. J Physiol. 2016;594(24):7419–33. doi: 10.1113/JP272885 ; PubMed Central PMCID: PMC5157065. 27730652 PMC5157065

[22] Yu T, Ferdjallah I, Elenberg F, Chen SK, Deuster P, Chen Y. Mitochondrial fission contributes to heat-induced oxidative stress in skeletal muscle but not hyperthermia in mice. Life Sci. 2018;200(1):6–14. Epub 2018/03/03. doi: 10.1016/j.lfs.2018.02.031 . 29499282

[23] Ganesan S, Summers CM, Pearce SC, Gabler NK, Valentine RJ, Baumgard LH, et al. Short-term heat stress causes altered intracellular signaling in oxidative skeletal muscle. J Anim Sci. 2017;95(6):2438–51. doi: 10.2527/jas.2016.1233 . 28727070

[24] Rogeri PS, Gasparini SO, Martins GL, Costa LKF, Araujo CC, Lugaresi R, et al. Crosstalk Between Skeletal Muscle and Immune System: Which Roles Do IL-6 and Glutamine Play? Front Physiol. 2020;11:582258. Epub 20201016. doi: 10.3389/fphys.2020.582258 ; PubMed Central PMCID: PMC7596683. 33178046 PMC7596683

[25] Dohl J, Passos MEP, Foldi J, Chen Y, Pithon-Curi T, Curi R, et al. Glutamine depletion disrupts mitochondrial integrity and impairs C2C12 myoblast proliferation, differentiation, and the heat-shock response. Nutrition research. 2020;84:42–52. Epub 20200919. doi: 10.1016/j.nutres.2020.09.006 . 33189431

[26] Cruzat VF, Rogero MM, Tirapegui J. Effects of supplementation with free glutamine and the dipeptide alanyl-glutamine on parameters of muscle damage and inflammation in rats submitted to prolonged exercise. Cell Biochem Funct. 2010;28(1):24–30. doi: 10.1002/cbf.1611 . 19885855

[27] Chen Y, Yu T, Deuster P. Astaxanthin Protects Against Heat-induced Mitochondrial Alterations in Mouse Hypothalamus. Neuroscience. 2021;476:12–20. Epub 2021/09/21. doi: 10.1016/j.neuroscience.2021.09.010 . 34543676

[28] Islam A, Deuster PA, Devaney JM, Ghimbovschi S, Chen Y. An Exploration of Heat Tolerance in Mice Utilizing mRNA and microRNA Expression Analysis. PLoS One. 2013;8(8):e72258. Epub 2013/08/24. doi: 10.1371/journal.pone.0072258 ; PubMed Central PMCID: PMC3744453. 23967293 PMC3744453

[29] Dohl J, Foldi J, Heller J, Gasier HG, Deuster PA, Yu T. Acclimation of C2C12 myoblasts to physiological glucose concentrations for in vitro diabetes research. Life Sci. 2018;211:238–44. Epub 2018/09/27. doi: 10.1016/j.lfs.2018.09.041 . 30253137

[30] Iqbal S, Hood DA. Oxidative stress-induced mitochondrial fragmentation and movement in skeletal muscle myoblasts. American journal of physiology Cell physiology. 2014;306(12):C1176–83. doi: 10.1152/ajpcell.00017.2014 ; PubMed Central PMCID: PMC4059998. 24740540 PMC4059998

[31] Yu T, Dohl J, Wang L, Chen Y, Gasier HG, Deuster PA. Curcumin Ameliorates Heat-Induced Injury through NADPH Oxidase-Dependent Redox Signaling and Mitochondrial Preservation in C2C12 Myoblasts and Mouse Skeletal Muscle. The Journal of nutrition. 2020;150(9):2257–67. Epub 2020/07/22. doi: 10.1093/jn/nxaa201 . 32692359 PMC7919340

[32] Hu H, Dai S, Li J, Wen A, Bai X. Glutamine improves heat stress-induced oxidative damage in the broiler thigh muscle by activating the nuclear factor erythroid 2-related 2/Kelch-like ECH-associated protein 1 signaling pathway. Poult Sci. 2020;99(3):1454–61. Epub 2020/03/03. doi: 10.1016/j.psj.2019.11.001 ; PubMed Central PMCID: PMC7587763. 32115031 PMC7587763

[33] Petry ER, Dresch DF, Carvalho C, Medeiros PC, Rosa TG, de Oliveira CM, et al. Oral glutamine supplementation attenuates inflammation and oxidative stress-mediated skeletal muscle protein content degradation in immobilized rats: Role of 70kDa heat shock protein. Free radical biology & medicine. 2019;145:87–102. Epub 20190907. doi: 10.1016/j.freeradbiomed.2019.08.033 . 31505269

[34] Phanvijhitsiri K, Musch MW, Ropeleski MJ, Chang EB. Heat induction of heat shock protein 25 requires cellular glutamine in intestinal epithelial cells. American journal of physiology Cell physiology. 2006;291(2):C290–9. Epub 20060322. doi: 10.1152/ajpcell.00225.2005 . 16554407

[35] Nissim I, States B, Hardy M, Pleasure J, Nissim I. Effect of glutamine on heat-shock-induced mRNA and stress proteins. J Cell Physiol. 1993;157(2):313–8. doi: 10.1002/jcp.1041570214 . 7901225

[36] Osborne JO, Stewart IB, Beagley KW, Borg DN, Minett GM. Acute glutamine supplementation does not improve 20-km self-paced cycling performance in the heat. Eur J Appl Physiol. 2019;119(11–12):2567–78. Epub 20190930. doi: 10.1007/s00421-019-04234-2 . 31565753

[37] Fuchs BC, Perez JC, Suetterlin JE, Chaudhry SB, Bode BP. Inducible antisense RNA targeting amino acid transporter ATB0/ASCT2 elicits apoptosis in human hepatoma cells. American journal of physiology Gastrointestinal and liver physiology. 2004;286(3):G467–78. Epub 20031016. doi: 10.1152/ajpgi.00344.2003 . 14563674

[38] Tu S, McStay GP, Boucher LM, Mak T, Beere HM, Green DR. In situ trapping of activated initiator caspases reveals a role for caspase-2 in heat shock-induced apoptosis. Nat Cell Biol. 2006;8(1):72–7. Epub 20051218. doi: 10.1038/ncb1340 . 16362053

[39] Kobayashi D, Watanabe N, Yamauchi N, Okamoto T, Tsuji N, Sasaki H, et al. Heat-induced apoptosis via caspase-3 activation in tumour cells carrying mutant p53. Int J Hyperthermia. 2000;16(6):471–80. doi: 10.1080/02656730050199322 . 11129259

[40] Rogero MM, Tirapegui J, Pedrosa RG, Pires ISO, Castro IA. Plasma and tissue glutamine response to acute and chronic supplementation with L-glutamine and L-alanyl-L-glutamine in rats. Nutrition research. 2004;24(4):261–70. doi: 10.1016/j.nutres.2003.11.002

